# Research Progress of Graphene Nano-Electromechanical Resonant Sensors—A Review

**DOI:** 10.3390/mi13020241

**Published:** 2022-01-31

**Authors:** Shang-Chun Fan, Yang Lu, Peng-Cheng Zhao, Fu-Tao Shi, Zhan-She Guo, Wei-Wei Xing

**Affiliations:** 1 School of Instrumentation Science and Opto-Electronics Engineering, Beihang University, Beijing 100191, China; lu_yang@buaa.edu.cn (Y.L.); zhaopc@buaa.edu.cn (P.-C.Z.); futaoshi@buaa.edu.cn (F.-T.S.); xingweiwei@buaa.edu.cn (W.-W.X.); 2 Key Laboratory of Quantum Sensing Technology, Ministry of Industry and Information Technology, Beijing 100191, China

**Keywords:** single-layer graphene sheet (SLGS), resonators, molecular structural mechanics, non-local elastic theory, molecular dynamics

## Abstract

Graphene nano-electromechanical resonant sensors have wide application in areas such as seawater desalination, new energy, biotechnology, and aerospace due to their small size, light weight, and high sensitivity and resolution. This review first introduces the physical and chemical properties of graphene and the research progress of four preparation processes of graphene. Next, the principle prototype of graphene resonators is analyzed, and three main methods for analyzing the vibration characteristics of a graphene resonant sheet are described: molecular structural mechanics, non-local elastic theory and molecular dynamics. Then, this paper reviews research on graphene resonator preparation, discussing the working mechanism and research status of the development of graphene resonant mass sensors, pressure sensors and inertial sensors. Finally, the difficulties in developing graphene nano-electromechanical resonant sensors are outlined and the future trend of these sensors is described.

## 1. Introduction

As the core device in measurements, silicon micro-resonant sensors play an important role in atmospheric parameter measurement system, aerospace ground test system, industrial automation production, consumer electronic products, etc. [1,2,3,4,5]. These sensors are featured by a resonant state of sensitive elements modulated by measurement, and by direct output of frequency signals to realize stable and repeatable measurements [6,7,8,9,10,11]. Limited by the properties of silicon, the size of traditional silicon microstructure sensors cannot be further reduced; otherwise, material failure will be caused. Therefore, it is difficult for these sensors to accurately measure weak force changes for space exploration, biomedical measurement, and ultra-micro-fine processing. This problem can be addressed by the birth of new materials, which has facilitated sensor development at a nanometer level. An outstanding new material is graphene with excellent structural and electromechanical properties. Nowadays, newly developed resonators have used graphene as sensor sensitive materials to realize physical quantity detection.

**Figure 1:**
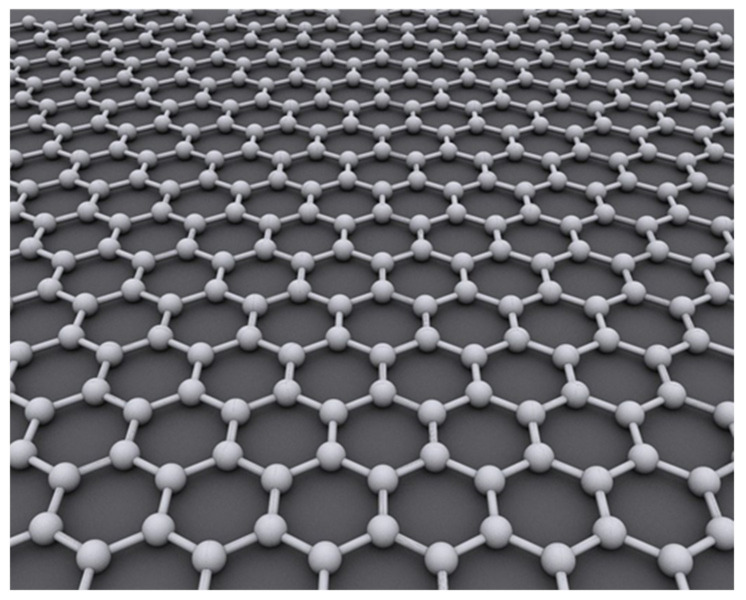
Graphene two-dimensional honeycomb structure.

In 2004, Konstantin Novoselov and Andre Geim of Manchester University used mechanical exfoliation to exfoliate a single-layer graphene sheet (SLGS) from graphite [12], triggering a wave of research on graphene materials and their applications. As shown in Figure 1, graphene is a two-dimensional honeycomb crystal, composed only of carbon atoms [13]. Due to this unique crystal structure, graphene has excellent electromechanical and mechanical properties. Research has shown that at present an SLGS is the thinnest material with a thickness of only 0.335 nm [14]. Its Young’s modulus is up to 1 TPa, and its breaking strength is about 130 GPa [15], 100 times more than that of stainless steel and far superior to the overload capacity of silicon, carbon nanotubes and other materials [16]. The density of graphene is about 2200 kg/m^3^, which is lower than that of single crystal silicon. When graphene has a size of micron, its fundamental frequency can reach megahertz [17]. In recent years, scholars have carried out in-depth theoretical and experimental research on the mechanical, electrical [18,19,20,21], optical [19,22,23,24,25,26], thermal [27] and chemical properties of graphene [28,29], predicting its application potential for high-performance sensors [30,31,32,33,34,35,36,37,38].

Because graphene has excellent crystallinity, the preparation process of single-layer and multi-layer graphene has been rather mature. There are mainly four preparation methods: micromechanical exfoliation [12], SiC epitaxial growth [39,40], chemical reduction [41,42,43], and Chemical Vapor Deposition(CVD). CVD is currently the most effective method for producing large-area graphene sheets with a guaranteed thickness. The graphene sheets produced in batches have the same excellent mechanical and electrical properties as those obtained by micromechanical exfoliation. The preparation process of CVD is compatible with the current integrated circuit process, and has become the mainstream process for preparing graphene devices [16,44,45,46,47,48,49], and the defects still need to be detected [50,51].

The present review focuses on the application of graphene in nano-electromechanical resonators. Herein, the birth of graphene and its physical and chemical properties are introduced, and the principle prototype of graphene resonators is presented. Three theoretical model of graphene sheets: molecular structural mechanics, non-local elastic theory and molecular dynamics. The preparation of graphene resonators is reviewed, followed by the application of graphene resonant mass, pressure and inertial sensors. This paper highlights high-performance graphene resonators, paving the way for future research on graphene nano-electromechanical resonant sensors.

## 2. Principle Prototype of Graphene Resonator

**Figure 2:**
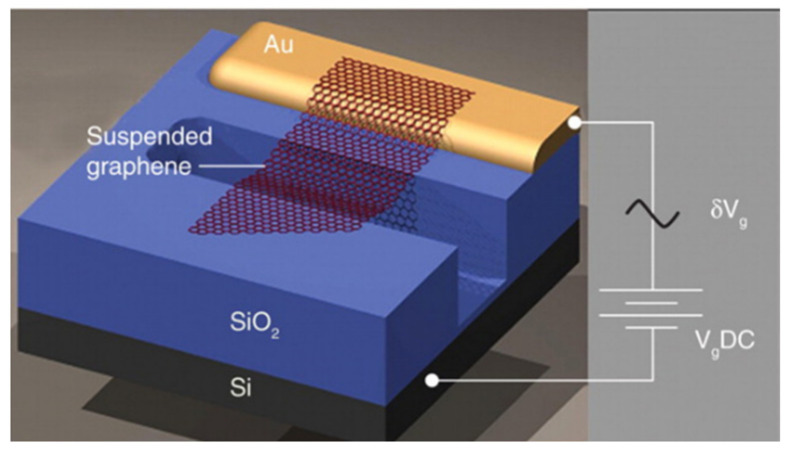
Prototype of graphene resonator [33].

After the emergence of the SLGS in 2004, the prototype of the nanoelectromechanical resonator is produced by Bunch with single-layer and multi-layer graphene sheets in 2007, providing an important experimental and theoretical basis for designing and examining graphene resonators (Figure 2). The single-layer or multi-layer graphene sheets obtained by mechanical exfoliation method is adsorbed on the etched SiO_2_ insulating layer with shallow grooves through intermolecular van der Waals force to form a double-clamped beam. Gold electrodes are arranged at both ends of the SiO_2_ layer, which can vibrate the resonator in MHz through electrical excitation. The frequency of a single-layer graphene resonator is 70.5 MHz, detected by optical interferometry, and the quality factor Q is about 78 in the experiment. This frequency is much larger than the resonant frequency when graphene is natural bending, indicating the presence of internal tension in the resonator. This result may be due to the friction between the graphene and the substrate during device preparation [33].

**Figure 3:**
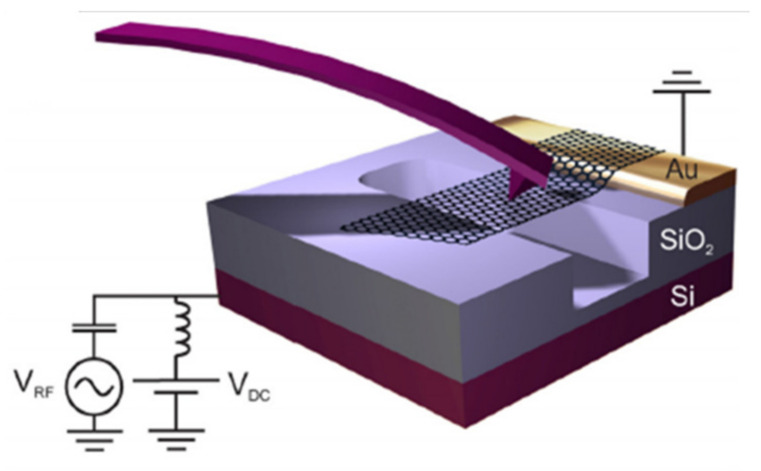
Schematic of the resonator together with the SFM cantilever [51].

In Figure 3, with using scanning probe microscope, the mechanical vibration of suspended graphene is detected, and the maximum vibration amplitude of the eigenmode occurred at the free edge rather than at the center of the graphene resonant beam. Finite element simulations show that this phenomenon is caused by the non-uniform stress inside the resonator, meaning that external vibration and corresponding tension must be considered in future experiments and applications. This finding indicates the direction for the future development of graphene tunable frequency resonators [51].

**Figure 4:**
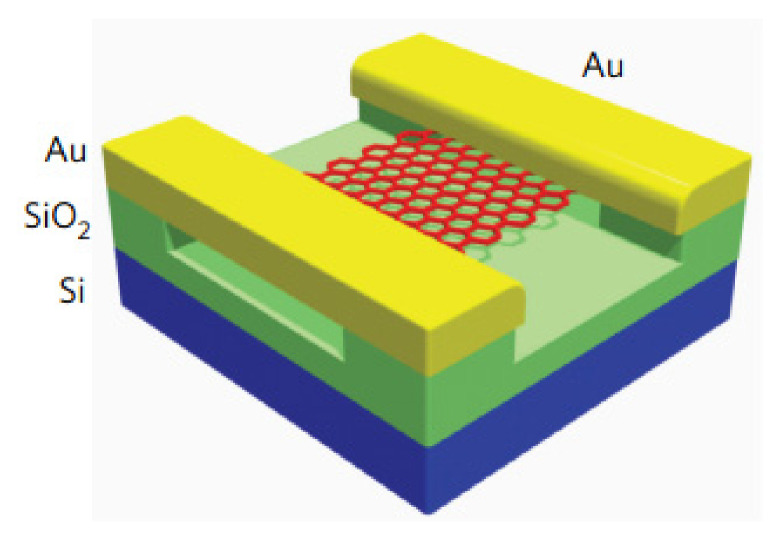
Schematic of single-layer graphene resonator model [52].

In 2009 a single-layer graphene resonator with electrical signal output is shown in Figure 4 [52]. The variation of the resonant frequency of the graphene resonator with additional mass, external temperature, silicon electrode voltage and other factors are tested. Experiments show that the resonant frequency and Q of graphene resonators increase as the temperature decreases, and that the frequency offset can be used to measure the thermal expansion coefficient of graphene. In addition, the Q value of the graphene resonator increases with the decrease of temperature (about 3000 at 100 K). Below 100 K, the energy dissipation of the resonator slows down, and Q can reach 10,000 at 5 K. Samples of different sizes, electronic properties and degrees of cleanliness all show almost the same results, but the reason for the effect of temperature on energy dissipation is not clear.

**Figure 5:**
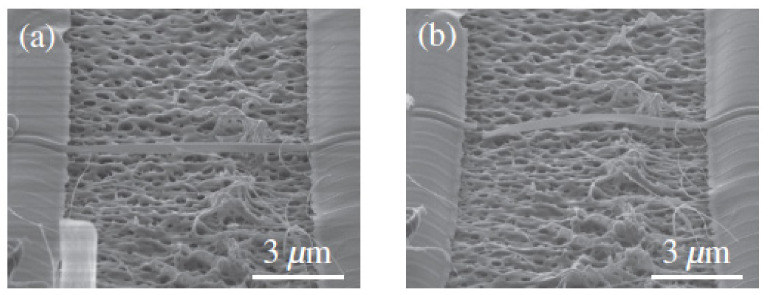
Graphene resonator with SU-8 resist substrate: (**a**) SEM image of graphene resonator after 500 °C annealing. (**b**) SEM image of broken graphene resonator after 600 °C annealing [53].

The resonator with suspended the graphene sheet on the surface of the SU-8 rubber substrate is shown in Figure 5, using the thermal expansion and contraction effect of SU-8 to change the tension of the graphene sheet [53]. Experiments show that the resonant frequency and Q value of the resonator increase to a certain extent after cooling. On the basis of this idea, a peripherally fixed drum resonator used SU-8 resist is fabricated [54]. Through experiments, it is found that the additional SU-8 resist doesn’t reduce the electrical properties of graphene, but increase its mechanical stiffness and improve the electromechanical response of the resonator.

The above explorations of the structural design, signal detection, and additional factors of the graphene resonator prototype have laid an important foundation for the subsequent application of graphene resonators in the field of sensing technology. In order to examine the resonant characteristics of graphene in depth, researchers have carried out comprehensive research from multiple perspectives such as the simulation and experiment of graphene resonant beams, graphene resonator processing, and applications of graphene resonators.

## 3. Vibration Characteristics of Graphene Resonant Sheet

Graphene resonators mostly use a single-layer graphene beam as sensitive element. The measured state can be reflected by the change of the output frequency of graphene beams. The thickness of single-layer graphene is only 0.335 nm. The establishment of the ultra-thin material graphene resonant beam model and the choice of analysis method affect the design of the graphene resonator structure and the experimental structural analysis. In this section, we will introduce three main analysis methods of graphene resonant sheets.

### 3.1. Molecular Structural Mechanics

Single-layer graphene is at the magnitude of microscopic atom and molecule. Molecular structural mechanics equates atoms to individual nodes. Using Cauchy-Born rule, the force between atoms is equivalent to that between nodes, and the vibration potential energy between atoms is converted to the strain energy in mechanics. Structural mechanical equations are established to obtain mechanical parameters, and molecular structural mechanics first needs to determine the model for graphene modeling.

In 2008, Juan et al. approximated a single graphene sheet to an elastic thin plate using the continuous elastic theory. The equilibrium shape and motion equation of the graphene sheet under external forces were derived. The static and dynamic responses of defect-free quadrilateral supported graphene were studied, and the formula for calculating the resonant frequency was deduced [55].

In 2009, Scarpa et al. proposed a truss-type analysis model and studied the in-plane linear elastic properties of graphene monolayers based on the theory of cell material mechanics. This study provids not only information about the mechanical properties of an SLGS, but also the results of the equivalent mechanical deformation mechanism when an SLGS is subjected to uniaxial small strain and pure shear load. Pure shear load exerts a tensile effect on the carbon-carbon bond of an SLGS [56]. In 2011, Georgantzinos et al. proposed a spring-based nonlinear finite element method to study the nonlinear mechanical behavior of graphene nanoribbons. An appropriate nonlinear spring was used to simulate the interaction between each atom, with the force-displacement curves of atoms following the potential energy differential equation of the bond deformation under the corresponding interaction. The results indicate that linear and non-linear mechanical properties depend on the chiral structure of graphene and the size of the graphene sheet [57]. In 2012, Rouhi et al. studied the buckling and vibration characteristics of the SLGS based on a cylindrical beam model. They regarded an SLGS as a spatial frame structure, which retains the discrete nature of graphene sheets, and used three-dimensional elastic beam elements to model the sheets. The critical compressive force and fundamental resonant frequency of the SLGS with different boundary conditions and geometric shapes are obtained and compared. The results show that the compressive buckling force decreases with the increase of the aspect ratio of a graphene sheet. At a low aspect ratio, an increase in aspect ratio results in a significant decrease in critical buckling load [58].

In 2013, Sakhacee et al. used molecular structural mechanics to simulate the vibration behavior of flawless SLGS at a constant temperature. The fundamental frequencies of the zigzag and armchair SLGS with different chiral configurations were obtained under the cantilever and fixed-support boundary conditions by simulating the carbon atoms in graphene lattices with equivalent structure beams and centralized mass units [59]. In 2013, Li et al. conducted a modeling analysis of the SLGS based on atomic potential energy. The graphene membrane, covalent bonds between atoms, and carbon atoms were considered as a frame-like structure, beam members, and nodes, respectively. The FE code of ANSYS commercial software was used to calculate the finite element model composed of beam elements, and the three-dimensional elastic Beam4 element was used for bonding modeling. The energy equivalence concept proposed by Rouhi and Anari was employed to establish the relationship between stiffness of beam elements in structural mechanics and force constants in molecular mechanics. The position of the mass attached to the graphene sheet and the influence of the different constraints of the sheet on the frequency and sensitivity of the resonator were analyzed [60]. In 2017, based on hexagonal network topology and single-electron tunneling model, Guo et al. proposed a single-electronic device network model based on graphene. The model describes the boundary constraints and the connection and coupling relationship matrix based on the graphene sensor. A simple expression of the detection molecule based on the graphene sensor is also introduced, which reduces the computational complexity of the model [61].

### 3.2. Non-Local Elastic Theory

Non-local elastic theory, an extension of classical continuum theory proposes that the conservation laws of mass, energy, and momentum are valid for an object as a whole, but that arbitrarily small voxels divided by the object may not hold. Additionally, the stress component of an arbitrarily small voxel as a reference point x depends on the strain components at position x, at all the other points of the object [62]. This theory can explain some atomic and molecular scaled phenomena that cannot be explained by continuum theory, such as high-frequency vibration and wave dispersion. Meanwhile, non-local elastic theory plays an important role in calculating of mechanical properties of components using graphene and carbon nanotubes as materials. This method has been widely used for graphene research.

In 2009, Murmu et al. studied the vibration characteristics of monolayer graphene sheets, using non-local elastic theory. The theory takes into account the small size effect introduced in the nanoscale structure. With a small size of an SLGS, it is found that the resonant fundamental frequency of an SLGS is rather sensitive to size changes [63]. In 2011, Wang et al. combined non-local slab model and molecular dynamics to study the vibration characteristics of single-layer and double-layer graphene sheets. The results show that the resonant frequency calculated by the classic graphene elastic model is relatively high, and that the small-scale effect, boundary conditions, and graphene size have a greater impact on the vibration characteristics of single-layer and double-layer graphene. The non-local slab model is important for the vibration analysis of graphene sheets with an edge length less than 8 nm [64].

In 2014, Chang et al. explored the possibility of using peripherally clamped double-layer circular graphene as a nano-mechanical resonator based on non-local elastic theory. The frequency equation of the resonant cavity with attached mass at any position was derived and the influence of the parameters on the vibration frequency and sensitivity of the resonator was studied. The results show that the farther the attachment mass is from the center of the resonator, the higher the frequency and the lower the sensitivity, that the frequency and sensitivity of the double-layer graphene resonator decrease with the increase of the aspect ratio, and that the sensitivity increases with the increase of the local nano-meter parameters [65]. In 2016, Shen et al. used the Galerkin method to derive the vibration frequency of the SLGS based on the non-local Kirchhoff plate theory [66]. In 2018, they used non-local theory to study the feasibility of a double-layer graphene sheet as a mass sensor resonator. These results show that the in-phase mode between single-layer graphene sheets and double-layer graphene sheets is similar. The sensitivity of the mass sensor can be improved by reducing the non-local parameters, making the attached nanoparticles close to the center of the double-layer graphene sheet and reducing the size of the graphene sheet. This finding is helpful for designing graphene mass sensors [67].

### 3.3. Molecular Dynamics

Molecular dynamics is an analytical method based on classical mechanics. The object of molecular dynamics is atoms, and this model is composed of several atoms. The core idea of molecular dynamics is to calculate the positions and trajectories of all atoms in the simulation system, using statistical analysis to obtain the macroscopic physical quantities of the system. Statistical physics holds that macro physical phenomena are intrinsically related to the movement of microscopic particles, and that the macroscopic quantity is equal to the statistical average of the microscopic quantities. Therefore, by knowing the relevant laws and trajectories of all atoms in the system, macroscopic physical quantities can be obtained.

Molecular dynamics examines atoms, and its model is composed of several atoms. The core idea of molecular dynamics is to calculate the positions and trajectories of all atoms in a simulation system, and obtain the macroscopic physical quantities of the system with statistical analysis. According to statistical physics, macro physical phenomena are intrinsically related to the movement of microscopic particles, and the macroscopic quantity is equal to the statistical average of microscopic quantities. Therefore, knowledge of the relevant laws and trajectories of all atoms in a system can generate macroscopic physical quantities.

**Figure 6:**
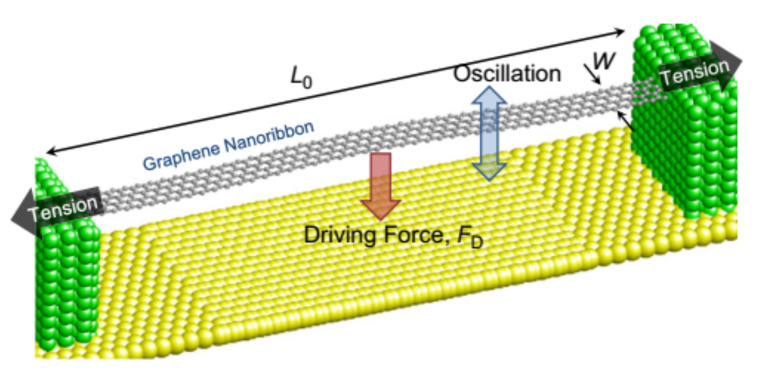
Graphene-nanoribbon-resonator [69].

With molecular dynamics, Kwon et al. studied a nano-scale graphene-nanoribbon-resonator in 2012, finding that the resonant frequency of graphene is greatly affected by driving force and axial strain, and that the tunable range of resonant frequency decreases exponentially with the increase of axial strain or driving force, as shown in Figure 6 [68,69]. Because most graphene bending deflection is in the elastic region of graphene, the function between resonant frequency and tension can be explained by classical continuum mechanics. The modeling technology used in these studies can be applied to other graphene-based nanoelectromechanical devices.

In 2013, Kang et al. studied suspended graphene nanoribbons resonators using molecular dynamics. The simulation results show that the resonant frequency of graphene nanoribbons depends on the average tension on both sides of the ribbons. The tension comes from two aspects. One is the deformation caused by the electrostatic force generated by the grid voltage; the other is the initial strain caused by the mismatch between the negative thermal expansion coefficient of graphene and the positive thermal expansion coefficient of the substrate when the ambient temperature changes. As the initial strain increases, the tunability and frequency tuning range of the graphene nanoribbons decrease. Therefore, if the axial tension can be changed by pending measurement, the graphene resonator will have the potential for the measurement of physical quantities such as high sensitivity mass, acceleration and pressure [70].

In 2013, Kim et al. used classical molecular dynamics simulation to study the mechanical response of nano-scale graphene resonators [71]. In 2014, Jiang et al. established a two-dimensional slab model, and analyzed the vibration characteristics of a graphene sheet using the finite element method [72]. In the same year, they used the finite element method to study the influence of the pressure difference between the inside and outside of the cavity on the four-side clamped graphene resonator, revealing the effect of the pre-tension and the number of layers on the resonant frequency and tunable range of the graphene resonator [73].

In 2016, Zhang used molecular dynamics simulation to study the vibration characteristics of an SLGS, including its free vibration resonant frequency and mode, and the effect of boundary conditions on the resonant frequency, such as size, initial strain, initial displacement, aspect ratio and chirality. It is found that the fundamental frequency of single graphene nanoribbons can reach GHz. When the size of graphene is small, there is a non-linear relationship between the resonant frequency and the size. With the increase of the size, the resonant frequency changes approximately linearly with the size. The strain change of graphene has greater influence on the fundamental frequency while the width and chirality of graphene have less influence on the change of the resonant fundamental frequency [74,75].

### 3.4. Summary

At present, there is no perfect theoretical model for the research on the resonant characteristics of graphene. Among the existing models, molecular dynamics is used to simulate the movement of molecules or atoms, and the result is closer to the experimental data. However, this method is usually used for nano-scale structure. For micron-sized structure, calculation is extremely time-consuming and complex, so it is necessary to find new solutions.

For multilayer graphene, macroscopic continuum theory can be used to approximate the model by using thin plate, shell and other continuous media models. Then the mechanical behavior and mechanical properties of graphene such as bending, stretching and vibration can be analyzed by Euler beam theory, Timoshenko plate and shell theory, etc. [76]. The numerical model can also be carried out by using commercial finite element analysis software such as COMSOL/ANSYS. However, the small-scale effect of an SLGS cannot be ignored. The semi-continuum model of graphene resonant beams proposed by Sun et al. can be used under specific conditions, which takes into account the dispersion characteristics of the material particles brought by the direction of graphene thickness entering the nanoscale [77].

The results of the above-mentioned studies show that for small size graphene sheets, the resonant frequency calculated by the classical continuous model is higher than that calculated by molecular dynamics or non-local elastic theory. This is because the small-scale effect of graphene is not taken into account by classical mechanical model. A comparison of molecular dynamics and non-local elastic theory shows that the small-scale effect of graphene depends on the boundary conditions and vibration modes of graphene, and that the small-scale effect has the least influence on the resonant frequency under the four-sided clamping. Thus, graphene nanoribbons with an appropriate size, aspect ratio, and boundary conditions are expected to be used to make high-sensitivity sensors detecting weak pressure, acceleration, and additional nano-mass.

## 4. Preparation of Graphene Resonators

The research of graphene resonator technology is the key to ensuring stable output of graphene resonators and their quantitative production. In recent years, the preparation process of graphene and graphene resonators has also been extensively studied.

**Figure 7:**
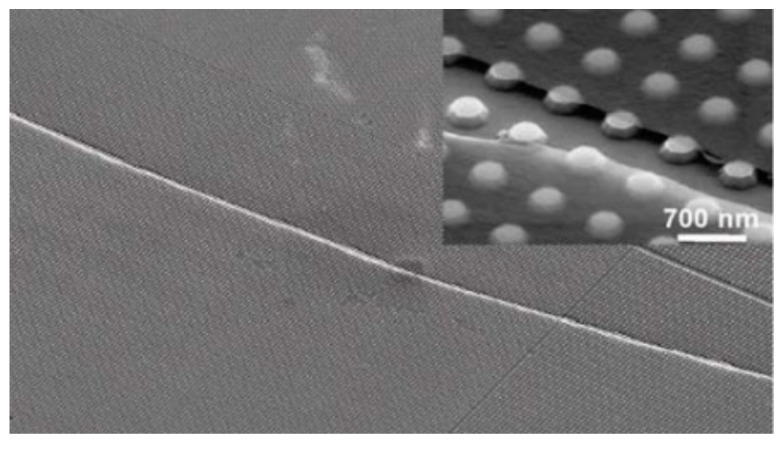
Transfer and drying of rGO(reduced graphene oxide) films in air on prepatterned substrates [78].

In 2008, Jeremy et al. pioneered the preparation of large-scale and few-layer reduced graphene oxide sheets through oxidation and exfoliation, and the drum-shaped graphene resonator is produced, as shown in Figure 7 [78].

**Figure 8:**
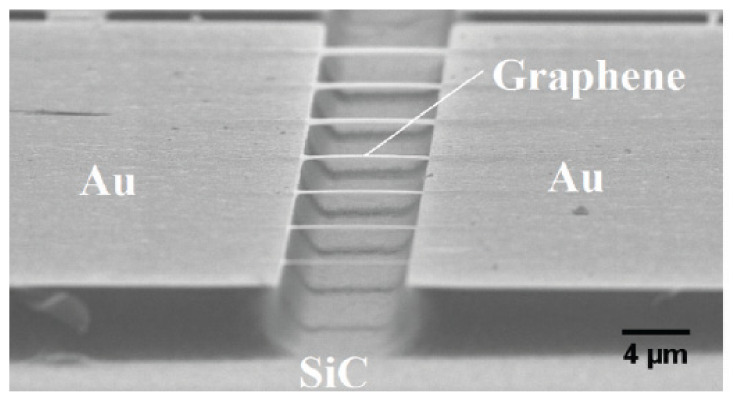
Double clamped graphene beam array [39].

A method is reported to fabricate a large-scale double-clamped suspended epitaxy single-layer graphene resonator by wet chemical etching, providing experimental and theoretical basis for the implementation of graphene resonator array. The size of the double-clamped graphene beam in Figure 8 is 8 μm × (0.5–3.5) μm [39]. To improve the transfer efficiency of large-area single-layer graphene grown on copper foil by CVD, reusable dry transfer and wet transfer technologies have been developed. The graphene can be transferred to substrates covered with shallow depressions, perforated substrates and flat substrates guaranteeing the integrity of the graphene, with fewer cracks and tears [79,80].

**Figure 9:**
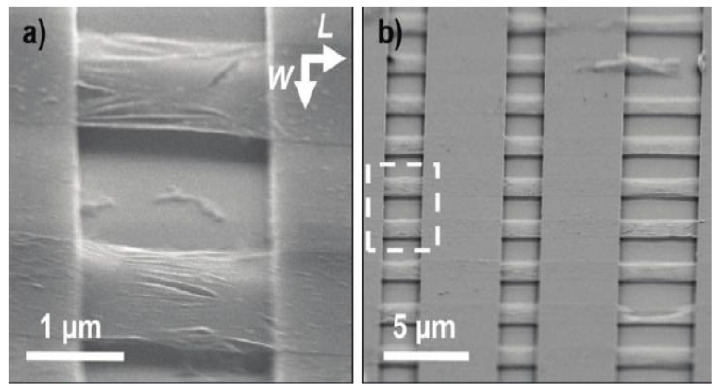
Suspended graphene sheet resonator array. (**a**) Angled scanning electron microscopy (SEM) image of Type A suspended graphene membranes over trenches in silicon oxide. (**b**) Angled SEM of an array of graphene membranes [81].

In 2010, Paul L. McEuen et al. produced large scale single-layer graphene sheets by CVD, and fabricated suspended graphene resonator array, as shown in Figure 9. The performance of the resonator was tested by electro-excitation and light detection. It was found that the graphene sheet resonator can be equivalent to a sheet with initial tension. The test results also show that the graphene sheets grown by CVD have the same excellent mechanical and electrical properties as the graphene sheets grown by mechanical peeling method, and that a large number of graphene sheets with the same properties can be produced by CVD method in batch [81].

The above-mentioned literature on the method for fabricating graphene resonators seldom discusses how to fabricate high-quality graphene sheets. In recent years, growing single-layer or multi-layer graphene on the surface of metal Cu and Ni foil or SiO_2_ film has been developed by CVD. The quality of the prepared graphene has been significantly improved, the size of the substrate is not limited, the cost is greatly reduced, and the transfer is easy to achieve [82]. The Institute of Physics of the Chinese Academy of Sciences studied the controllable epitaxy growth of graphene on metal surfaces. The epitaxy preparation process and structure characteristics of graphene on various metal single crystals (Ru, Pt, Ni, Ir, Cu, Pd, etc.) have been studied in detail [83]. Among these metal single crystals, a continuous defect-free SLGS with millimeter size and high quality has been obtained on the surface of Ru (0001) [84]. However, since etching is isotropic, the SiO_2_ under the metal electrode contact will be removed, resulting in mechanical structural instability in the process of using buffered hydrofluoric acid (BHF) to remove part of the SiO_2_. The manufacturing process of suspended graphene devices by using LOR etchant effectively solves this problem. The polymer under the graphene layer was effectively removed by using organic solvents irresponsive to inorganic materials such as metals and insulators, and a desirable effect and a stable mechanical structure have been obtained [85].

These preparation and transfer technologies have opened up possibilities for manufacturing various graphene devices with unique configurations and enhanced performance.

## 5. Application of Graphene Resonators in NEMS

As soon as the graphene resonator was developed, research on its application to sensors has been reported. As early as 2007, Bunch et al. pointed out the great potential of graphene resonators in quality and mechanical quantity detection during the experimental study of graphene nano-electromechanical resonant sensors (NEMS) [33]. In this section, the application of graphene in nano-electromechanical systems is reviewed. The development of three NEMS is introduced: graphene resonant mass sensors, pressure sensors and some inertial sensors.

### 5.1. Graphene Resonant Mass Sensor

The fundamental vibrational frequency of micron-level single-layer graphene has reached MHz [17], and any additional mass or adsorbed gaseous substance will change the vibration state of an SLGS inspires a new design direction in the detection field that requires sensitive extremely weak mass. Mass sensors based on graphene resonant nanoelectromechanical devices are expected to detect tiny particles such as bacteria (a single mass of about 10–12 g) and viruses (a single mass of about 10–15 g).

In 2008, using molecular structural mechanics, Sakhaee et al. studied the influence of single-point mass and atomic dust on the resonant frequency of an SLGS. The numerical results show that the SLGS is sensitive to adsorption mass, and that the sensitivity of mass detection is as high as 10–21 g. For the same size of the additional mass, the frequency offset of a graphene sheet increases with the increase of the length-width ratio of the sheet [59].

**Figure 10:**
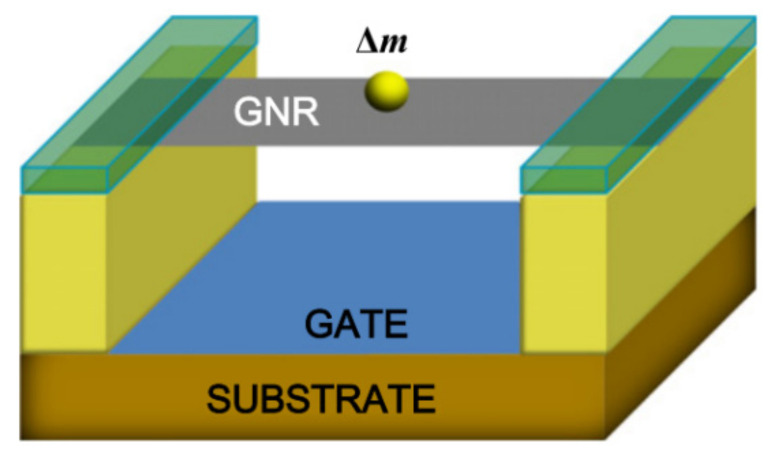
Schematic diagram of graphene resonator with additional mass [86].

In 2013, Kwon et al. used molecular dynamics simulation method to study the relationship between the fundamental frequency and additional mass of a double-clamped suspended graphene resonator, as shown in Figure 10. The additional mass ∆m deposited on the graphene sheet changes the equivalent mass Meq of the sheet, thereby changing the natural vibration frequency of the graphene sheet. The simulation shows that the double-clamped graphene resonator can achieve a mass resolution of 10–24 g. The frequency-mass response curve of the resonator reveals a strong linear relationship when the additional mass is in the range of 10–21~10–19 g. It should be noted that the molecular dynamics simulation used by Kwon et al. was carried out at ultra-low temperature of 1 K, without considering the effect of thermal diffusion on the vibration characteristics and measurement sensitivity of graphene sheets in the actual working environment [86].

In 2014, Fazelzadeh et al. studied a nano-size mass sensor based on an SLGS in a thermal environment, using rectangular nano-sheets simply supported on four sides as structural model. The non-local slab model and Galerkin numerical calculation method were used to derive the analytical solution of the frequency deviation of the SLGS with respect to variables such as nanoparticles (i.e., additional mass), temperature, and nanoparticle position. Numerical calculation shows that the relative frequency deviation of the SLGS increases with the increase of temperature, the sensitivity also increases. The closer the additional nanoparticles are to the center of the sheet, the greater the relative frequency shift becomes [87]. In 2015, Karličić et al. studied the influence of the in-plane magnetic field on the graphene resonant mass sensor. With non-local Kirchhoff theory, they analyzed the vibration characteristics of the SLGS, finding that with the increase of the external magnetic field strength, the vibration frequency of the sheet increases, and that the sensitivity of mass detection also increases [88].

**Figure 11:**
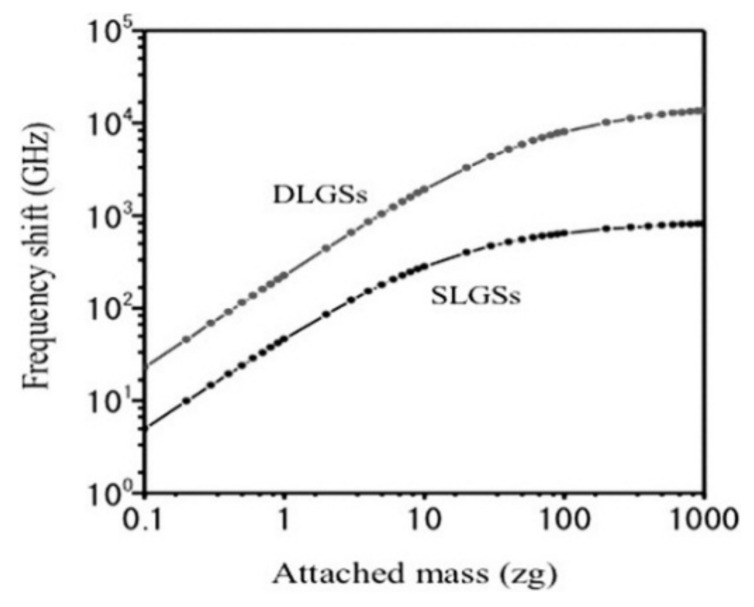
Comparison of frequency offset between double-layer and single-layer graphene sheets [89].

In 2013, Natsuki et al. studied the feasibility of simply supported double-layer graphene sheets for mass sensing. The frequency-additional mass response of single-layer and double-layer graphene sheets is compared and analyzed by the continuum model, as shown in Figure 11. It can be seen that, in the case of the same size and additional mass, the double-layer graphene sheet has a larger frequency deviation than the SLGS and has a wider logarithmic linear range. Therefore, compared with an SLGS, a double-layer structure can achieve higher measurement sensitivity; that is, it is easier to use the resonant frequency to calculate the size of the additional mass [89]. Other graphene resonant mass sensors have been designed by continuum model simulation. It is found that the graphene sheet has higher mass detection sensitivity in nonlinear vibration state, with the sensitivity affected by the initial in-plane tension of the graphene sheet [90].

In 2019, Xiao studied the relationship between the resonant frequencies of peripherally supported graphene sheets and the adsorption mass by molecular dynamics simulation. The effects of different high-order modes, the size and the position of the adsorption mass were studied, and a mass solution method based on high-order modes was proposed [91]. In 2021, the influence of the local mode of the double-clamped graphene sheet on mass sensitivity was studied. The different responses of the local and the basic modes to adsorption mass, and the effects of the local modes on the quality factor of mechanical vibration were examined. The results show that increasing the aspect ratio and pre-stressing of graphene sheets can effectively improve the quality factor of the graphene resonant mass sensor [92].

Taken together, single or double-layer graphene resonators have the potential for ultra-high sensitivity of mass detection. However, current research mainly involves theoretical analysis and simulation calculation, lacking necessary experimental data. This will pose difficulties for practical application of the graphene resonant mass sensor.

### 5.2. Graphene Resonant Pressure Sensor

Pressure sensors are one of the most widely used sensors in industry, and many physical quantities of these sensors are shown by pressure. Graphene is a sensitive element in graphene resonant pressure sensors, and has two main shapes: drum shape and beam shape. When subjected to pressure, graphene changes its resonant frequency, and detecting this change can help measure pressure. The difference of the theoretical model lies in the modal analysis of peripheral fixed support and double-clamped of graphene. Table 1 shows the comparison of the structure, analysis method and index of several graphene resonant pressure sensors.

**Figure 12:**
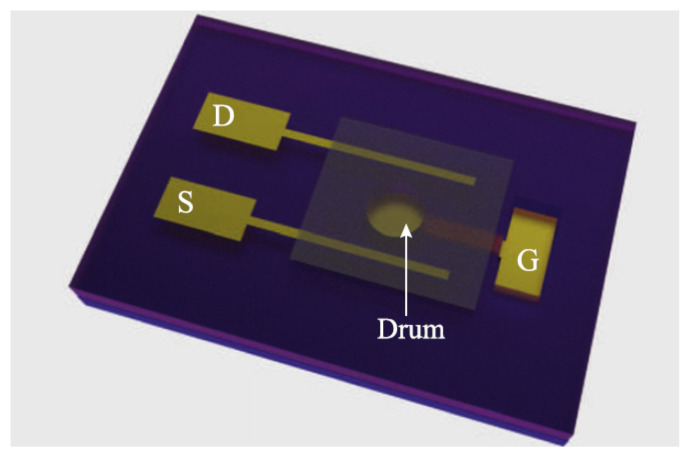
Drum-shaped graphene resonant pressure sensor [93].

**Figure 13:**
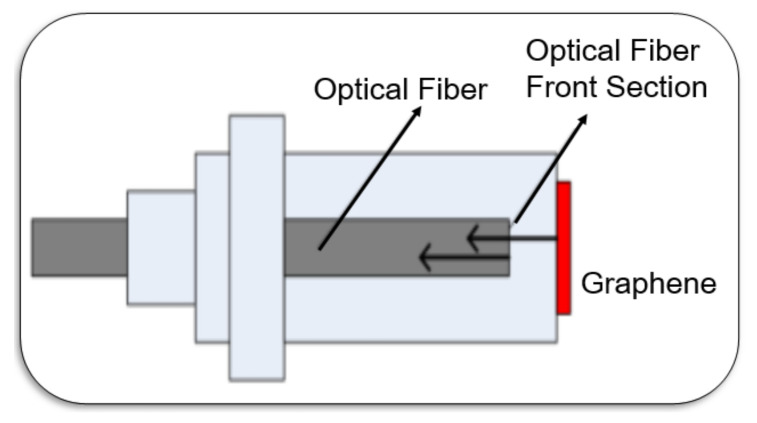
Fabry-Perot light cavity graphene resonant pressure sensor [95].

In the design of drum-shaped graphene resonant pressure sensors, Raj eliminated parasitic capacitance with high-resistance sapphire (resistivity > 1014 Ωm) substrate. Through direct radio frequency drive and detection, the resonant frequency of the multilayer graphene sheet becomes as high as 200 MHz and the mechanical quality factor is close to 500, as shown in Figure 12 [93]. The graphene pressure “micro-drum” sensor designed by Wang was realized by transferring large-area, multilayer graphene on a suspended silicon nitride film perforated by a periodic micro-pore array [94]. This study used the optical interference principle of the Fabry-Perot light cavity to detect the resonant frequency of the graphene sheet, and calculated the measured pressure value in Figure 13 [95].

**Figure 14:**
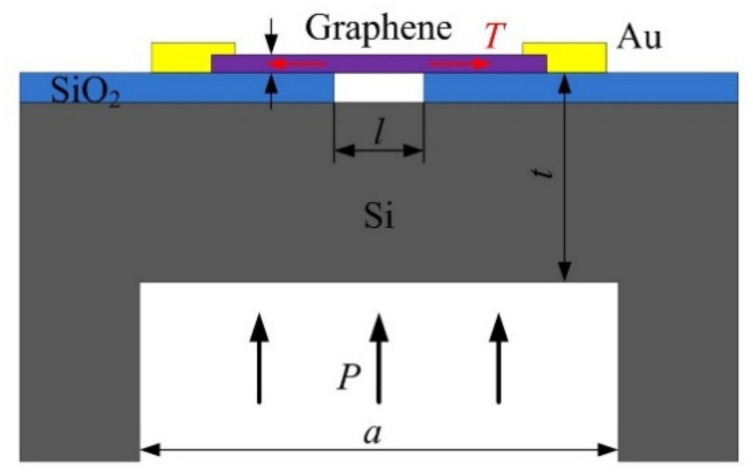
Double-clamped graphene beam resonant pressure sensor [96].

**Figure 15:**
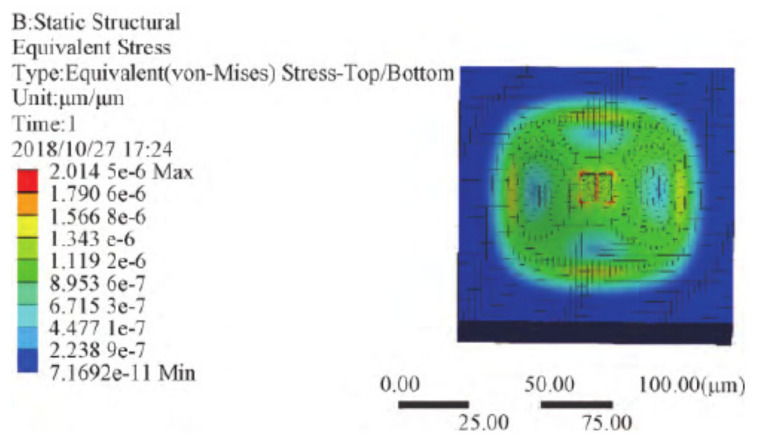
Single graphene beam resonant pressure sensor [97].

For graphene resonant pressure sensors with a double-clamped beam structure, the secondary sensitivity method has been mostly used to avoid direct contact between the graphene beam and the medium, mainly in the stage of finite element simulation. The structure designed by Jiang employed the concept of classic silicon micro-resonant pressure sensor compound sensitivity. The square silicon diaphragm was used as the primary sensitive element to directly sense the measured pressure. This pressure acted on the silicon diaphragm to deform the silicon diaphragm, causing the axial strain and stress change of the graphene beam, while the rectangular graphene beam suspended above the cavity acted as a secondary sensitive element to indirectly sense the pressure as shown in Figure 14 [96]. With the single graphene beam resonant pressure sensor in Figure 15 [97], Fan designed a dual-beam graphene resonant pressure sensor [98], which can realize differential detection and reduce common mode interference.

The experiment and simulation of the structure of graphene resonant pressure sensors enable the detection of a series of physical quantities that can be output through changes in force. On this basis, inertial force and Coriolis force have been effectively expressed.

### 5.3. Graphene Resonant Inertial Sensor

Inertia is the inherent property of all objects. They have inertia whether they are solid, liquid or gas, and whether they are moving or stationary. Inertial measurement is to detect the state of an object by using inertial sensitive elements such as gyroscope and accelerometer to determine the relative position and state of an object. This measurement is used in many fields such as navigation positioning, consumer electronics, and motion carrier control. Inertial sensors are divided into two categories: angular velocity gyroscope and accelerometer. The detection principle of the gyroscope is mainly based on: Coriolis effect and Sagnac effect. For Coriolis effect, Coriolis force is proportional to the input angular rate; for Sagnac effect, phase difference is proportional to the input angular rate. The detection principle of the accelerometer is mainly based on Newton’s second law, in which the magnitude of the acceleration of an object is proportional to the force and inversely proportional to the mass of the object, and the direction of acceleration is the same as that of the force. Graphene resonant inertial sensors can achieve high sensitivity signal output due to the mechanical property of graphene, but the structure design of the sensor itself is the basis for effective, reliable and high-quality signal detection.

After 2007, Bunch et al. examined graphene resonators, through experimental analysis, and proved that graphene has a strong adhesion to the surface of SiO_2_ substrate through intermolecular van der Waals force. This adhesion is also reflected in the proof mass of graphene to SiO_2_/Si, which can realize the adhesion of small mass at the bottom of suspended graphene [99,100,101]. Such characteristics make graphene sensitive to pressure changes, further realizing the sensitivity of graphene to inertia.

**Figure 16:**
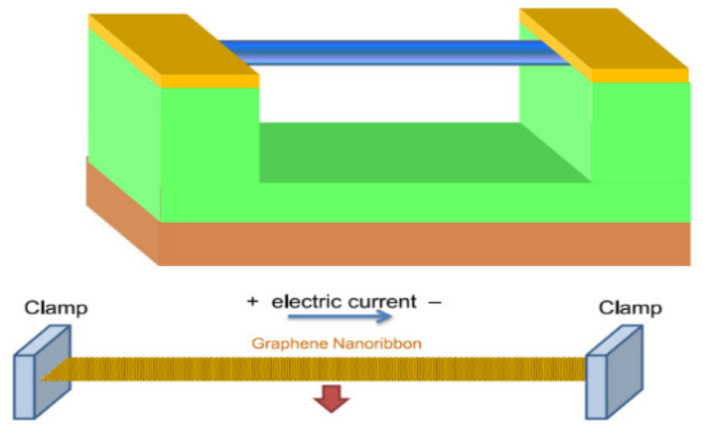
Schematic diagram of graphene nanoribbon acceleration sensor [102].

In 2012, Kang et al. designed a graphene nanoribbon acceleration sensor, as shown in Figure 16. In this design, graphene nanoribbons are suspended on the substrate and form an equivalent parallel plate capacitor with the substrate. The inertial force produced by external acceleration will change the mechanical vibration state of graphene nanoribbons, resulting in changes in the capacitance of parallel plate capacitor and the conductivity of graphene nanoribbons. The measured acceleration can be calculated by measuring the capacitance change or vibration frequency offset through the current oscillation circuit [102]. In 2015, Kang et al. studied the dynamic characteristics of accelerometers based on suspended cross graphene resonators through molecular dynamics simulation. It is found that the deflection motion of the transverse graphene resonator under the action of the driving force is similar to that of the graphene nanoribbon resonator [103].

**Figure 17:**
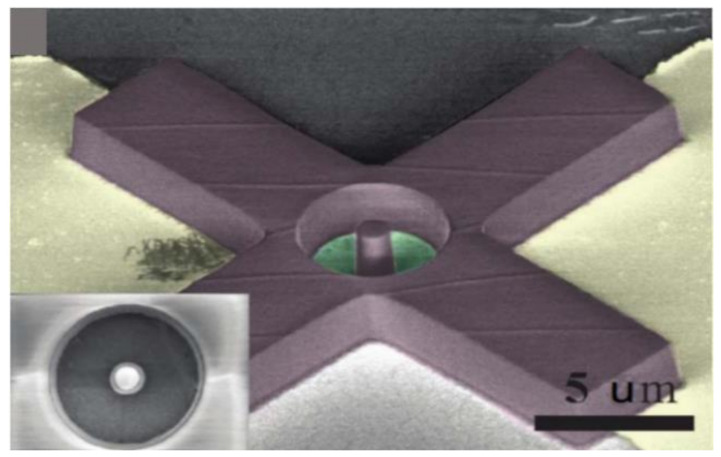
SEM image of an SU-8 resist graphene accelerometer [54].

In 2015, Lee et al. combined graphene drum resonator and SU-8 resist proof mass to create a graphene accelerometer, as shown in Figure 17. In this structure, the gate is generated on the silicon substrate by deep UV lithography and the gate surface is covered with a layer of the SiO_2_ insulating film. The layer is polished by CMP method and the graphene sheet is transferred on the layer, and then the periphery of the graphene film is fixed on the SU-8 resist substrate to form a drum resonator. On this basis, the cylindrical SU-8 resist proof mass is placed at the center of the graphene sheet. The proof mass senses the acceleration to change the deflection of the graphene sheet and change the conductivity of the graphene sheet. The acceleration value is obtained by measuring the change of conductivity. Although the detection method is non-resonant, the preparation method of drum resonator provides a reference process route for preparing graphene resonant accelerometer [53,104].

**Figure 18:**
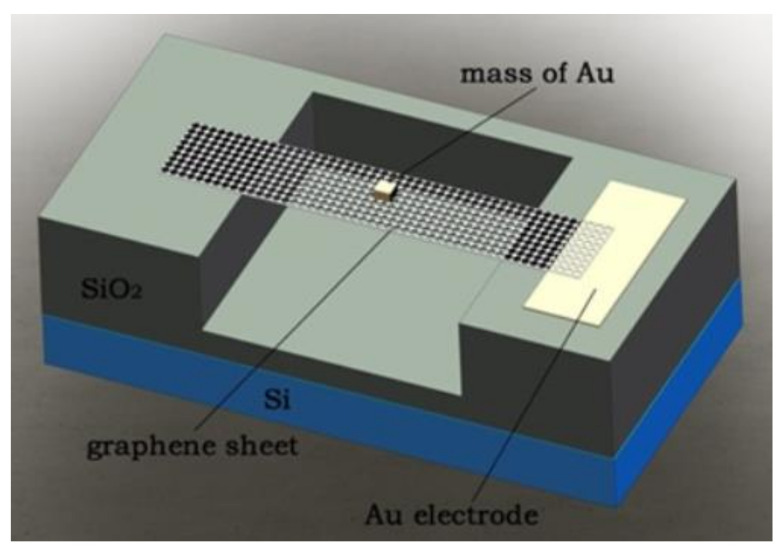
Model of graphene acceleration sensor [105].

In 2017, Jie et al. simulated and analyzed the sensitivity of the graphene resonator to acceleration by using COMSOL Multiphysics finite element software. The sensitive structure is shown in Figure 18. The rectangular graphene sheet with mass block constitutes a double-clamped resonator, and the graphene sheet is suspended above the U-shaped SiO_2_ insulating layer. Under the action of external acceleration, the additional mass senses the inertial force and causes the deformation of the graphene sheet, resulting in the change of the resonant frequency of the graphene sheet [105]. In 2018, Shi et al. designed the structure of differential graphene resonant accelerometer through COMSOL, and calculated the differential mode in double beam working states. This study improves the acceleration detection sensitivity and measurement accuracy and suppresses conjugate interference [106].

In the design of a graphene resonant acceleration sensor, sensitive mass block is usually introduced as a sensitive element for acceleration detection to change the vibration state of graphene. This introduction enables graphene to generate residual stress during the transfer of graphene to the mass block. In 2020, Fan uses SOI wafer to release the process flow of mass block by sacrificially removing the box layer, combining dry etching with steam HF etching. The weight of sensitive mass block Si is three orders of magnitude greater than that of SU-8 [107], six orders of magnitude greater than that of gold [108], and seven orders of magnitude greater than that of carbon [104]. The weight of suspended mass block increases significantly, which has potential significance for miniaturized NEMS inertial sensor applications [47].

In 2021, Lu et al. designed a graphene resonant gyroscope structure with direct output frequency. The variation law of resonant frequency of graphene with different structure parameters and angular velocities is calculated by finite element simulation. The ultra-high sensitivity of diagonal velocity can realize the sensitivity to extremely weak angular velocity [109].

In summary, the graphene resonator can realize the detection of mass, force and other physical quantities based on the measured modulation law of the vibration characteristics of graphene sheets. The preparation technology of graphene materials and related resonators has achieved great progress and has made corresponding progress in the fields of quantum effects and optoelectronic devices [23,24,25,26,37]. However, the current research on graphene resonant sensing technology is in the stage of theoretical analysis and numerical simulation, and there is still a lack of sufficient literature reports on the overall structural design and related experimental research of the sensor.

## 6. Conclusions

This paper has reviewed the properties of single-layer graphene, four common graphene material preparation technologies, and the development of graphene resonators from the perspectives of experiments, vibration characteristic analysis and preparation process. This review focuses on the application of graphene resonators in mass, pressure and inertial measurements. Since the research on graphene nano-electromechanical resonant sensors is still in its infancy, two main challenging problems remain to be solved before the fabrication of graphene resonant sensor products:

(1) Process. It is still difficult to prepare high-quality graphene with a regular shape, but it is easy to introduce initial stress and strain in the process of graphene matrix transfer. This affects the tunability of graphene resonators. In addition, the effect of electrostatic force, temperature and other interference factors should be fully considered.
(2) Overall structure design. The existing literature mainly studies the effect of sensing on graphene resonators from the perspective of the graphene resonator itself, but the research related to the overall structure modeling and optimization analysis of the sensor is not sufficient. A perfect structure is helpful in improving the measurement sensitivity and measurement range of the sensor, and is thus convenient for the design of device processing technology. Overall structure design of graphene resonant NEMS sensors needs to adequately consider the reasonable processing technology, boundary conditions and actual working conditions, reasonably using the theoretical and simulation analysis methods.

In addition, the graphene resonant sensor works in a resonant state, so the realization of closed-loop self-excited system and weak signal detection are also the key to technical issues. Graphene resonant sensor cannot replace MEMS sensor at the current level of processing and detection

To sum up, graphene plays an important role in the field of nano-electromechanical resonators, and graphene resonators has developed rapidly due to their high quality with preparation optimization and high sensitivity detection. In the future, graphene nano-electromechanical resonant sensors will have broader prospects in such fields as biological detection, energy and chemical industry, and inertial navigation.

## Figures and Tables

**Table 1:** Structure comparison of graphene resonant pressure sensor.

Graphene Shape	Sensitive Method	Analysis Method	Sensitivity Analysis	Year/Author
Drum Shape	Pressure Direct Sensitive	Experimental Verification	1Torr	2016 Raj [93]
	Pressure Direct Sensitive	Experimental Verification	2.8 × 10^−5^ mbar^−1^	2016 Qiugu Wang [94]
	Pressure Direct Sensitive	Experimental Verification	0.298 kHz/kPa (10~1000)kPa	2016 She [95]
Beam Shape	Secondary sensitive	Finite Element Simulation	26.838 kHz/kPa (0–1000)kPa	2014 Jiang [96]
	Secondary sensitive	Finite Element Simulation	81.3 Hz/Pa (0–10) kPa	2016 Fan [97]

## Data Availability

The data is included in the main text.
